# RGB-D SLAM: A Review of Methods and Performance Trade-Offs for Different Requirements

**DOI:** 10.3390/s26113513

**Published:** 2026-06-02

**Authors:** Yixin Yan, Jinling Li, Qiuyang Liu, Siqian Lv, Qing Wu

**Affiliations:** The Higher Educational Key Laboratory for Measuring & Control Technology and Instrumentation of Heilongjiang Province, Harbin University of Science and Technology, Harbin 150080, China

**Keywords:** RGB-D SLAM, performance trade-off, deep learning, multi-sensor fusion

## Abstract

The RGB-D (Red, Green, Blue plus Depth) camera is a widely used onboard sensor for Simultaneous Localization and Mapping (SLAM), as it captures RGB images and depth information simultaneously. This capability has made RGB-D SLAM a topic of broad research interest. We present a comprehensive introduction to the framework of RGB-D SLAM and illustrate representative approaches in each component. Furthermore, we examine the conflicts among diverse performance metrics and discuss performance trade-offs in cutting-edge RGB-D SLAM from a requirement perspective, as achieving a SLAM system with perfect performance is challenging in the present landscape. This manuscript merges theory and practice, serving as a reference for the selection of RGB-D SLAM methods under various practical applications and offering a structured perspective for future development of RGB-D SLAM.

## 1. Introduction

Simultaneous Localization and Mapping (SLAM) has been utilized in the realm of Autonomous Driving [1], Augmented Reality [2], Robotics [3], Industrial Automation [4], Unmanned Aerial Vehicle [5], etc., broadly. A key characteristic of SLAM is that it relies only on onboard sensors to perceive the surrounding environment and achieve high-quality pose estimation and mapping without prior information or external signals [6]. Additionally, loop closure enables the system to recognize the true topological structure of environments and resist drift, thereby building a globally consistent map [7]. As a consequence, SLAM is well-suited for unknown and challenging environments where external signals may be unreliable, which has contributed to its widespread use. While SLAM approaches share collective characteristics and orientations, there is still considerable diversity among them. Especially in terms of sensor types, SLAM methods are primarily segmented into Visual SLAM and Light Detection and Ranging (LiDAR) SLAM [7]. LiDAR SLAM is widely regarded as a mature technique, and its recent progress has been relatively slow [8]. By contrast, the rapid advancements in deep learning and computer vision endow Visual SLAM, classified as Monocular SLAM [9], Stereo SLAM [10], and RGB-D SLAM in accordance with the camera type, with novel vitality.

RGB-D SLAM, which uses an RGB-D camera and inherits the advantages of SLAM, is characterized by a promising method and has been researched widely since gaining the capacity to obtain depth information directly [11] and low expenditure. RGB-D cameras appeared in the early 2000s but were not as widely used as modern commercial products because of technical defects and high costs. They were typically specialized equipment found in research laboratories or industrial applications. Kinect sensors, introduced by Microsoft Research in 2011, were a significant milestone in the popularization of RGB-D camera technology and getting the research of RGB-D SLAM off the ground. KinectFusion was the first real-time 3D reconstruction system based on an RGB-D sensor, demonstrating the feasibility of combining depth sensing technology with iterative closest point (ICP) algorithms to generate detailed 3D models of indoor environments in real time. More recently, GPS-SLAM [12], a real-time 3D reconstruction system hybridizing a colorized signed distance field with 3D Gaussian splatting, achieves 150+ fps on Azure Kinect sequences—delivering an order-of-magnitude speedup over existing dense RGB-D SLAM methods while maintaining comparable reconstruction quality. However, the system suffers from a lack of loop closing and struggles in large-scale environments. Nadia Figueroa et al. used a combined approach toward consistent reconstructions based on 6D RGB-D Odometry and KinectFusion by feeding the estimated pose to the KinectFusion algorithm [12]. Additionally, Felix Endres et al. presented a mapping system that robustly generates highly accurate volumetric 3D maps of the environment for robot localization, navigation, and path planning using an RGB-D camera [13]. The approach is evaluated quantitatively and achieves an accuracy of 9.7 cm and 3.95° with a frame processing time of 0.35s on average. Furthermore, in 2014, they presented a beam-based error evaluation model (EEM) to evaluate the quality of a frame-to-frame estimate [14]. By rejecting highly inaccurate estimates, the approach enables a mapping system based on an RGB-D camera to deal with challenging scenarios robustly. In recent years, RGB-D SLAM has further advanced in research, achieving high levels of real-time performance, robustness, accuracy, and various performance metrics. In addition, because of the capacity to create a dense environment map, RGB-D SLAM is widely used in the semantic VSLAM direction.

**Figure 1:**
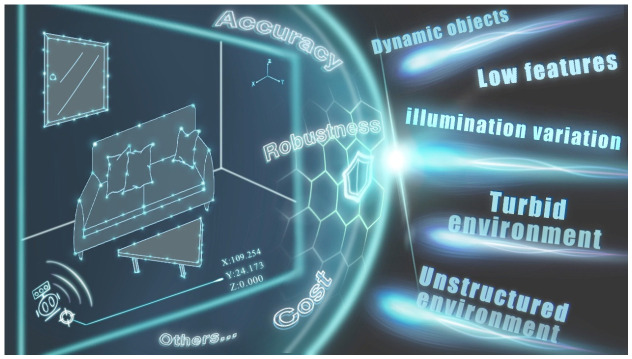
The conceptual framework of RGB-D SLAM facing requirements.

However, it is universally acknowledged that RGB-D SLAM is not just an endeavor as a research thesis but also an application issue. In the context of applications, the requirements are no longer in pursuit of high accuracy or real-time independence but mandate the designer of the system to consider the performances, tasks, environments, etc., simultaneously. This intricate combination problem is unable to be ideally addressed, resulting from the natural paradox between different performances such as accuracy and real time. Hence, the objective of RGB-D SLAM, as with all SLAM methods, transforms into striving to achieve a balance for the requirements in applications. Of special note is that the adjustable range of the point is determined by the performance metrics required for the task, and the ability to keep the point in the range is the robustness of the system. The advantages of RGB-D SLAM facilitate resisting the outward impact, whereas the disadvantages of RGB-D SLAM, comprising limited depth measurement range, limited perception range, large resource footprint, motion blur, etc., will be magnified under this condition. Dynamic objects [15], light fluctuation [16], and high vehicle velocity [17] pose robustness challenges for the SLAM system. It will render failure if the balance point is beyond the range after being impacted. Accordingly, various strategies (e.g., sensor fusion [18], feature extraction [19], dynamic object removal [20], back-end optimization [21], and mapping [22]) have been applied to alleviate these problems and maintain the necessary balance for task completion (Figure 1). Nevertheless, RGB-D SLAM techniques have not yet been systematically summarized, which motivates this review.

In this manuscript, we explore the state of the art in RGB-D SLAM and review classic frameworks and recent achievements in terms of requirements, demonstrating the trade-off between performances catering to specific scenes. The remainder of this review, which refers to RGB-D SLAM, is structured as follows: Initially, we introduce the core components of the RGB-D SLAM framework, ranging from sensors to the mapping module. State-of-the-art RGB-D SLAM approaches are elaborated on the basis of the manner of sensor fusion. Ultimately, a conclusion is drawn, and the advancement trend of RGB-D SLAM is discussed. It provides a preliminary insight for new researchers and can be used as a reference when leveraging RGB-D SLAM to solve practical problems.

## 2. The Framework of RGB-D SLAM

**Figure 2:**
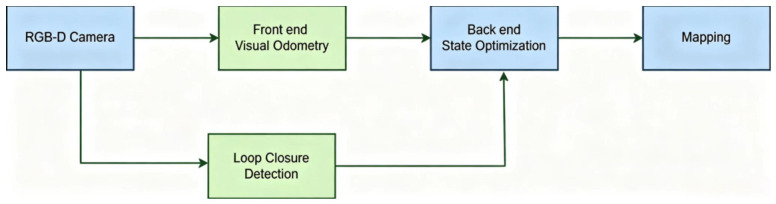
The framework of RGB-D SLAM.

The integrated framework of RGB-D SLAM consists of five modules, including sensors, front end, back end, loop closing, and mapping, in a general manner, as depicted in Figure 2. Sensors, at the core of the RGB-D camera, are responsible for perceiving the outward environment and detecting the state of the system itself. The information channeled by sensors is processed into models suitable for localization and mapping in the front end. For the purpose of enhancing the accuracy of pose estimation and the consistency of mapping to the maximum extent, on one hand, the back end precisely estimates and updates the pose and map through optimization algorithms based on the outcome of the front end [20]. On the other hand, the cumulative error can be lessened substantially by means of loop closing. Eventually, the mapping module achieves mapping to the back for follow-up missions.

These five modules form the typical closed loop of RGB-D SLAM. The choice of the sensor directly affects data quality in the front end, while the coordination among modules determines the overall performance trade-off.

### 2.1. RGB-D Camera

**Figure 3:**
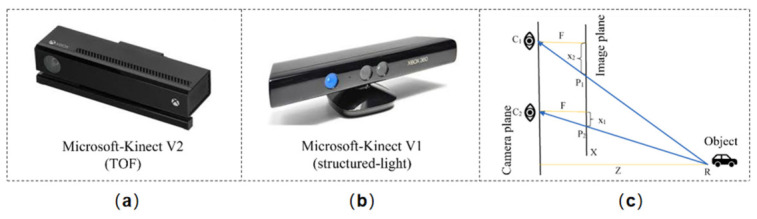
(**a**) The Microsoft Kinect V2 sensor, (**b**) RGB-D camera, (**c**) Schematic diagram of a stereo camera calculating depth information. Reproduced from ref. [26].

The RGB-D camera is critical for the whole RGB-D SLAM system and has the capacity to furnish RGB images and depth information [3]. The integration of color and depth data enables the system to solve scale drift with less complexity and paves the way for the construction of dense 3D maps [23]. A succession of experiments, taking sensor specifications into account, has been conducted by Imad El Bouazzaoui et al., and the results demonstrate that RGB-D camera acquisition modes play an important role in SLAM systems and impact accuracy [24]. RGB-D cameras can be categorized into two types in light of the underlying principles of measuring depth information: the time-of-flight (TOF) camera and the structured-light camera. Figure 3a,b depict the products of two kinds of RGB-D cameras. The former obtains depth information by means of calculating the time delta between the emission of the light signal, typically infrared, and the reception of the returned light signal [25]. The latter acquires depth information by projecting a pattern of light, like stripes or a grid, onto objects and analyzing the deformation of the pattern via triangulation when it strikes surfaces [25]. In a comparison view, time-of-flight cameras have the merits of high measurement speed, light resistance, and wide measurement range, while structured-light cameras have reduced hardware costs, high accuracy, and spatial resolution. Consequently, for application, time-of-flight cameras are suited to scenarios that require high real-time performance and no exacting requirement for accuracy. When utilized in outdoor environments, the power must be sufficient to resist the potential interference from natural lighting conditions. By comparison, a structured-light camera is preferable for precise measurements within small-scale indoor environments but struggles in high-intensity lighting and outdoor environments.

It is worthwhile mentioning that stereo cameras are capable of getting depth information at the expense of lengthy processing [9] (Figure 3c). Apart from poor real-time performance, the effectiveness of stereo cameras diminishes with texture-less surfaces and over long distances, which is a formidable issue for large-scale and featureless environments. Table 1 demonstrates a comparison between the three types of cameras.

TOF RGB-D cameras offer balanced performance and the lowest price, whereas stereo cameras provide the longest range but are far more expensive. Structured-light RGB-D cameras excel in short-range, low-power indoor modeling, though their price is higher than that of TOF cameras.

In addition, the calibration of RGB-D cameras is indispensable for fulfilling the requirements for high accuracy in practical applications. By refining intrinsic parameters, including focal length and lens distortion, and aligning extrinsic parameters to coordinate the RGB and depth sensors, calibration ensures that the depth information corresponds precisely with the visible image. Some notable devices have been presented to calibrate the parameters of RGB-D cameras recently. Filippo Basso et al. introduced a calibration framework applied to estimate intrinsic and extrinsic parameters of RGB-D cameras via the usage of a novel two-component error model [25]. Hongyan Liu et al. leverage a sphere-based calibration framework to achieve the calibration of intrinsic and extrinsic parameters [25]. Luis-Rogelio Roman-Rivera et al. used an RGB-D camera calibration strategy, which simplifies the calibration protocol [25]. In the multiple RGB-D Cameras cases, Alejandro Perez-Yus et al. proposed a method, based on line observations, to calibrate the extrinsic parameters [27]. The calibration of the RGB-D camera is beneficial for RGB-D SLAM in a taxonomy of applications. In summary, TOF and structured-light cameras differ in range, accuracy, and power consumption; calibration further compensates for systematic sensor errors, laying the foundation for subsequent front-end processing.

### 2.2. Front End of RGB-D SLAM

The front end of RGB-D SLAM, also known as Visual Odometry (VO), is responsible for furnishing the motion information of the RGB-D camera for mapping and achieving preliminary localization based on interframe estimation of input RGB images and depth images [28]. It is deemed as a subsystem of RGB-D SLAM without optimization and loop closing to lean on [8], therefore, in the following discussion, Visual Odometry and RGB-D SLAM system will not be distinguished. The performance of the front end of RGB-D SLAM influences various aspects of the system in terms of accuracy, real time, and robustness directly, so the significance is important. The novelty and modification of the front end is still an active discussion and one of the main thrusts behind the advancement of RGB-D SLAM hitherto.

Since RGB-D cameras are capable of providing high-quality, high-resolution RGB-D images with fine details, low-light scenes, or environments with many particles haunting in the cloud, the post-processing in the front end [29]. Motivated by the inaccuracy and high failure rate in challenging environments where the output images of RGB-D cameras are underexposed or blurry, image enhancement and image restoration have served as an extra component in the front end to enhance the availability of images channeled by RGB-D cameras, thereby improving the effect of data association to a certain extent [9]. Cho Younggun and Kim Ayoung presented a real-time and channel-invariant visibility enhancement method leveraging a hybrid image enhancement method in 2018 [16]. Shohei Mori et al. presented an incremental RGB-D inpainting approach dubbed InpaintFusion applicable not only to normal but also to non-planar scenes [30]. Under various experiments, InpaintFusion performs satisfactorily, is globally consistent, and provides proinpainting results via the integration of fusion and multi-keyframe inpainting. Additionally, neural networks have been employed in image enhancement and image restoration. Yizhuo Quan et al. estimate pixel-wise grayscale transformation curves via a three-dimensional convolutional neural network to enhance images in low-light environments [31]. Ziqiang Zheng et al. proposed a generative adversarial network (GAN) to perform high-fidelity image enhancement [32]. It is worth mentioning that Javier Navarrete et al. presented a color smoothing approach to accelerate RGB-D image production and elevate image quality by capitalizing on entropy information [33].

**Figure 4:**
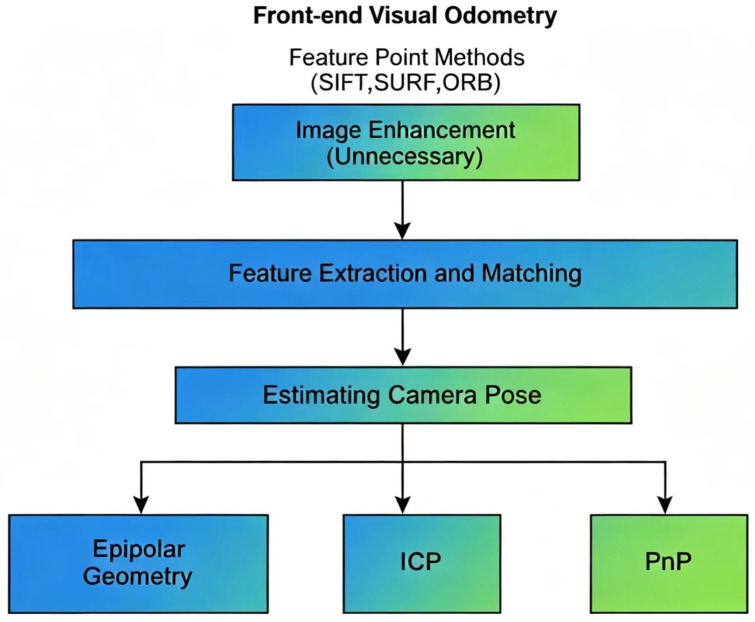
Process of feature point method.

With the aid of image enhancement and image restoration, RGB-D cameras have a boosted capacity of providing high-quality images in most cases. However, using all image details results in an overwhelming amount of data and impairs the real-time performance of the system, which is hard to accept in practical applications. To reduce the computational burden, feature-based methods have become a mainstream strategy. Their feature extraction process essentially offers a way to decrease computation by selecting informative features from images. The process is shown in Figure 4. Features, which are pixels that share common attributes and differ from adjacent pixels, must be invariant to rotation, orientation, translation, scaling, and luminous intensity in the context of RGB-D SLAM [34]. The majority of point features detection and matching methods of RGB-D SLAM comprise the Scale Invariant Feature Transform (SIFT) [35], Speeded Up Robust Features (SURF) [35], Oriented FAST (Features from Accelerated Segment Test) and Rotated Binary Robust Independent Elementary Features (BRIEF), which together form the ORB (Oriented FAST and Rotated BRIEF) descriptor [35]. Substantial experiments manifest that SIFT is accurate at extracting features, but has the drawback of considerable calculation [36]. Accordingly, SURF takes integral images to accelerate feature detection on the foundation of SIFT. In this regard, ORB, the combination of the FAST feature detector and the BRIEF descriptor, is comparatively fast, enabling real-time performance. [37]. Besides point features, other types of features (e.g., lines, planes [38], surfels [39], edges, and combinations thereof [40]) have been applied to the front end of RGB-D SLAM. These features and their corresponding descriptors lay a firm basis for addressing the data association problem [41]. The features extracted from the current frame are matched with features in previous frames to identify feature pairs [42]. The matched features are associated with landmarks or specific objects in the scene for subsequent pose estimation utilizing Triangulation, Epipolar geometry, Perspective-n-Point (PnP), etc., followed by the camera trajectory obtained by continuously estimating the motion of the camera. Feature Slam [43], a real-time camera tracking system based on 3D Gaussian splatting, was proposed by Thirgood et al. in 2026. The VO defensibly interpolates motion results adaptively, and a comprehensive objective function is applied to select key frames in a deterministic method. Concurrently, within the trajectory accumulation, the matched features are projected into three-dimensional space to construct a map depicting the motion of the camera.

**Figure 5:**
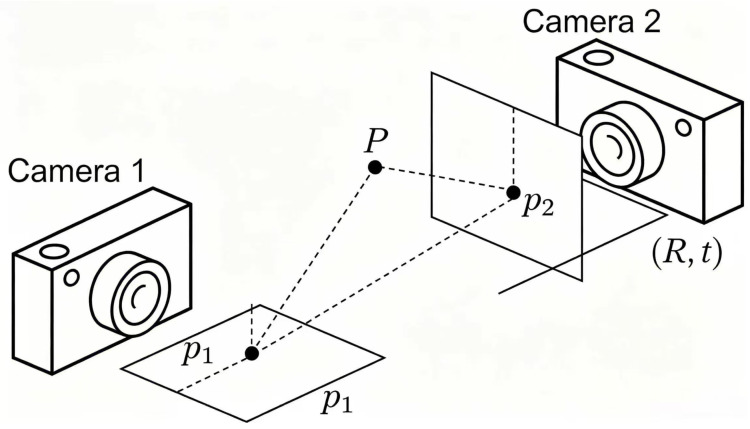
Schematic diagram of the photometric error in the direct method.

Regardless of the popularity of the feature-based front-end method, there are still concerns around discarding most of the information in the image. In contrast, the direct method operates directly on pixel intensity, allowing for the preservation of all information contained in the image [44]. The continuous sequence of images captured by the camera is used to construct the inter-frame optical flow field, and then leverage the optical flow field and motion parameters to calculate pixel motion constraints for each pixel, which are used to establish the motion model of the camera. Direct methods fully utilize the brightness information of images [45] and circumvent the need for feature extraction and matching. Consequently, it exhibits superior computational efficiency compared to indirect methods and also demonstrates strong adaptability to environments featuring dynamic scenes and low-texture surroundings [46]. The principle of gray-level invariance is shown in Figure 5.

Additionally, integrating deep learning facilitates the implementation of RGB-D SLAM in intelligence requirements. The usage of semantic information enables the system to recognize the scene more effectively, which performs preferably in terms of insensitivity to illumination changes, distinguishing dynamic objects, and feature matching compared with geometric information [41]. Deep learning, as an effective strategy, is leveraged in RGB-D SLAM at large [26] and has a future direction. Thus, front-end methods have evolved from feature-based to direct and then to semantic deep learning, each offering a different trade-off among real-time performance, robustness, and information utilization.

### 2.3. Back End of RGB-D SLAM

The back end realizes the state optimization on the foundation of the front end to achieve accurate pose estimation and map [44] and its algorithms are mainly classified into filter-based methods and optimization-based methods [8]. The former principally harnesses the Bayesian principle and estimates the current state, merely relying on the state at the previous time step and current observation data [47]. RGB-D SLAM employing filter-based optimization methods is still valuable. Jiazhao Zhang et al. leverage Particle Filter Optimization, accelerated by updating a particle swarm template, to achieve per-frame optimization at high speed without inertial information. Furthermore, these particles are evaluated utilizing a depth-only approach to obtain robust and accurate pose tracking under rapid camera motion [48]. Other representative filter-based methods, including the Extended Kalman Filter (EKF) [49] and Unscented Kalman Filter (UKF), etc., are also utilized in SLAM systems. However, filtering-based methods are problematic when dealing with motion models that exhibit strong non-linearity, and the storage capacity of the covariance matrix increases exponentially with the size of the state vector, limiting their applicability to large scenes. Predominantly, RGB-D SLAM systems use graph optimization. The graph optimization method involves representing the back-end optimization algorithm as a graph [50], where the vertices represent the poses of the subject and environmental features at different time instances, and the edges represent the constraint relationships between these vertices. The optimization-based algorithm is applied to solve the pose of the camera after the graph is built and the state is optimized on the vertices to meet the constraints on the corresponding edges, preferably [51]. While considering all the historical data requires high storage capacity, graph optimization is also a formidable issue. In short, filtering is efficient but less effective under strong non-linearity, whereas graph optimization is more accurate but demands higher storage and computation; actual systems must choose according to task resources.

### 2.4. Loop Closure Detection

The pose estimation appears to have localization errors stemming from sensor errors, feature matching inaccuracies, etc., and the error accumulates with the increase in operation time [8] thereby influencing the global consistency of motion trajectory estimation and mapping. Loop closure detection meets the need of evacuating cumulative error by identifying whether the camera revisits the same position. Namely, loop closure detection is a feedback mechanism in SLAM that aims to achieve a globally consistent map [8]. Correct loop closure detection not only substantially improves mapping accuracy but also enables the system to recognize the true topological structure of the scene [52]. BoW (Bag-of-words) leverages words to represent the features in the scene and detects loop closure by counting the number of the same words between images, which is a mainstream method [53,54]. Images are represented by just one vector in BoW, thereby reducing computational and storage burden strikingly [55]. Hash-based strategies [56], which utilize a global image descriptor, detect loop closure by means of comparing the hash values of images, and have fast processing times. Deep learning exhibits a clear capacity for differentiating and clustering features and has been exploited for loop closure detection to tackle the challenge of multifarious feature categories. Zhou and Sun propose a loop closure detection method based on a lightweight Siamese capsule network, which extracts capsule-based feature vectors and computes image similarity via cosine distance, achieving enhanced robustness under illumination and viewpoint changes [55]. Additionally, Huang Li et al. presented a semantic loop closure method, and the detection accuracy increased by the combination of semantic and geometric information [57]. Whether BoW, hashing, or deep learning is in use, loop closure detection always balances recognition efficiency and reliability to support global consistency.

### 2.5. Mapping

The quality of the generated map directly impacts the robustness and accuracy of the SLAM system. High-quality maps enable systems to operate effectively in navigation, obstacle avoidance, and environmental reconstruction [58]. Maps can be segmented into metric maps and topological maps [8] on the basis of representation methods. Generally, the metric map can be further divided into sparse maps [59] and dense maps [60]. The metric map delineates the positional relationships among map elements, whereas the topological map highlights the connectivity relationships. Research on RGB-D SLAM Mapping modules has improved in accuracy and real-time performance. Lichao Xu et al. built a 2D Occupancy Grid Map, using a 2D mapping module, for the original ORB RGB-D SLAM to improve the capacity of real-time location of VSLAM [61]. The method achieves marker position measurement accuracy ranging from 0.039 m to 0.186 m, and marker distance measurement accuracy ranging from 0.018 m to 0.235 m. By means of representing the environments as a collection of overlapping SDF submaps, Victor Reijgwart et al. presented a globally consistent, lightweight building approach dubbed voxgraph, which is capable of being equipped on computationally constrained devices [62]. Additionally, the problem of insufficient high-level intelligence mapping can be solved under human remote guidance. A human–robot collaborative 3D mapping framework was introduced by Jianhao Du et al., which evaluates camera pose estimation via a binary hypothesis testing [63]. The semantic map, as an appealing method, boils down to the semantic information, enabling a system equipped with perceptual ability. Ehsan Zobeidi et al. proposed a GP regression method so that the robot team is capable of building dense metric-semantic maps based on streaming RGB-D observations [64]. From metric to topological maps, and from sparse to dense to semantic maps, the evolution of the mapping module reflects different emphases on storage cost, geometric detail, and task intelligence.

### 2.6. Relocalization

Notably, occlusions, rapid camera motion, sudden changes of viewpoint, etc., result in the presence of camera tracking failure, and relocalization is committed to lessening the blow of it [21]. Ben Glocker et al. utilize randomized ferns to realize keyframe encoding, thereby presenting an RGB-D Camera relocalization approach in real time [65]. Ruihao Li et al. proposed a dual-stream neural network that is trained by stages to realize indoor relocalization, and an RGB-D image encoding approach has been exploited in the neural networks [66]. Experiments show that using this approach, the relocalization accuracy increased by 20% compared with cutting-edge deep learning methods, and the system gets a better handle on tackling environmental changes. Jikai Wang et al. realize visual relocalization via the Regression Forest algorithm, utilizing Coarse-to-fine thought, based on RGB-D images input [67]. Tommaso Cavallari et al. modified regression forests to evade the flaw of needing to be trained in the target environment by leveraging a pre-trained forest and utilizing a relocalization cascade to achieve accurate RGB-D camera pose estimation without sacrificing real-time performance [68]. Relocalization plays a pivotal role in maintaining reliable localization performance, thereby ensuring the consistency of mapping. Relocalization serves as a key fault-tolerance mechanism; its accuracy and real-time performance are a typical trade-off, and deep learning methods are gradually narrowing this gap.

## 3. State-of-the-Art RGB-D SLAM

### 3.1. Feature-Based Method

#### 3.1.1. Point Features

Point features are a mainstream avenue of RGB-D SLAM, attributed to the wide range of ORB-SLAM2. ORB-SLAM2 [8], presented by Raul Mur-Artal and Juan D. Tard’os, is a significant contribution to the scope of SLAM and is one of the most widely used open-source feature-based frameworks in RGB-D SLAM currently. ORB-SLAM2 supports three types of cameras: monocular, stereo, and RGB-D, demonstrating excellent versatility. ORB-SLAM2 is composed of three main parallel threads, including tracking, local mapping, and loop closing. The front-end feature detection utilizes ORB descriptors, which balance efficiency and accuracy, and the objective of ORB-SLAM2 is long-term and globally consistent localization. Consequently, ORB-SLAM2 achieves great real-time performance and does not require GPU processing. It facilitates the implementation of SLAM in practical applications and lays solid foundations for follow-up studies. Regarding the back-end, bundle adjustment (BA) is leveraged for accurate trajectory estimation, and the results demonstrate that bundle adjustment outperforms photometric and depth error minimization or ICP in RGB-D cases. Additionally, a lightweight localization mode enables the system to realize accurate localization without drift and with relocalization capability. In spite of having satisfactory real-time performance and accuracy, even delivering the capacity of resisting dynamic objects to some extent, RGB-D SLAM still has intractable issues that cloud its scope of application and require improvement.

**Figure 6:**
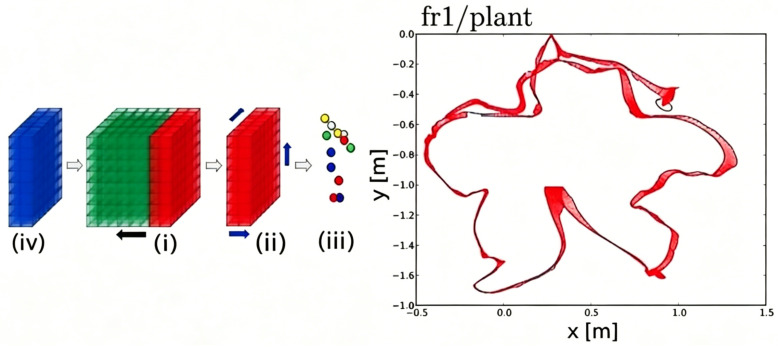
The process of volume shifting and trajectory in large-scale environments. Labels (**i**–**iv**) mark distinct phases along the estimated camera path: (**i**) initial camera pose at the start of the sequence, (**ii**) the point where volume shifting begins (e.g., the volumetric fusion window moves to follow the camera), (**iii**) a location where loop closure is detected and constraints are applied, and (**iv**) the final pose after global optimisation. These markers help to visualise how the system manages large-scale mapping by dynamically shifting the volume and closing loops [70].

Jing Yuan et al. presented ORB-TEDM on the basis of ORB-SLAM2 [69]. The primary characteristic of the approach is to leverage the combination of triangulation estimates and depth measurements provided by an RGB-D sensor to reliably achieve precise 3D position estimates of feature points, thereby producing a consistent map. ORB-TEDM has a drift of 1.814 m, whereas ORB-SLAM2 has 3.071 m in a real corridor environment. In order to circumvent the limitation of the lengthy process, Qiang Fu et al. presented a descriptor-independent keypoint matching approach so that the system tracks keypoints without computing descriptors [69]. In addition, RGB-D SLAM is considered limited in indoor environments. Thomas Whelan et al. presented a SLAM system [70] using an RGB-D camera, which creates dense globally consistent maps over hundreds of meters in real time. The approach first extends the volumetric fusion of depth maps, then utilizes geometric and photometric constraints to realize camera pose estimation, and finally, updates the dense map via place recognition and subsequent loop closure constraints. The process of volume shifting and trajectory in large-scale environments is depicted in Figure 6. This valuable work set the stage for RGB-D SLAM in large-scale environments.

Cesar Cadena proposed that the research on SLAM needs to put more emphasis on robustness [44]. One primary issue influencing robustness is dynamic objects in dynamic environments, and it is inevitable in practical applications [71]. The static world assumption affirms the significance of removing dynamic objects. In response, researchers have presented several solutions, and the approaches to cope with dynamic environments flourished thereafter. To overcome the degeneration caused by dynamic objects, Yuxiang Sun et al. introduced a motion removal strategy via pixel segmentation of the foreground [72]. This strategy requires no prior information about the moving objects. The method is capable of augmenting the front end of the SLAM system as a pre-processing stage. The average Root Mean Square Error (RMSE) improvements from the proposed method for the Absolute Trajectory Error (ATE), translational drift, and rotational drift in highly dynamic environments on the TUM dataset are 85.63%, 78.08%, and 71.22%, respectively. Ran Long et al. presented a dense RGB-D SLAM approach that can detect and track each of the multiple dynamic objects, even occupying the scenario at large independently [73]. Regretfully, it cannot evade the flaws of the overlap of dynamic objects. Moreover, an RGB-D SLAM approach proposed by Ran Long et al. titled RigidFusion [74], in contrast to the conventional method, which copes with dynamic objects as outliers or tracking, depends on prior information. The key characteristic of the method is that the dynamic component is assumed to be rigid, and the dynamic and static portions are tracked at the same time. As a result, the system enables tracking and reconstruction of even the dynamic objects occluding over 65% of the scene, and with 17 cm/s speed. Yu Liu et al. harnessed a dynamic feature point detection method based on double K-means clustering and established static weight, composed of static probability and a static observation number, for each feature point to resist the presence of dynamic objects and augment the accuracy of pose estimation [75]. To evaluate the confidence of static feature points, the static probability graph (SPG) and the static observation number (SON) are established. The proposed method demonstrates a clear improvement in accuracy of 94.36% compared to ORB-SLAM2 and 20.83 fps real-time performance.

A few articles lessened the blow of dynamic objects reliant on assigning the static points and dynamic points. Xin Yang et al. proposed a robust and highly accurate RGB-D SLAM [75] for dynamic scenes, which can be equipped on a central processing unit, delivering substantial improvement in terms of pose estimation in experiments with a trivial increase in processing time of 16.6 ms per frame. A dynamic keypoint exclusion approach, the core of the system, has been exploited to resist pose estimation errors caused by dynamic objects. Additionally, the approach has great portability, which paves the way for other keypoint-based VSLAM. Detecting dynamic objects has the possibility of misidentification by taking advantage of short-time span frames under a low-dynamic environment scene. SGDO-SLAM presented by Hu et al. in 2025 [75] estimates the initial camera pose via robust feature matching and distinguishes dynamic from static components using a coarse-to-fine dynamic rejection strategy combined with static weighted optimization. In contrast to other systems that detect dynamic features using short time-span frames, DFR-SLAM capitalizes on a long period of keyframes and demonstrates remarkable ATE RMSE improvement in highly dynamic environments, with an improvement of over 92%. Similarly, Zheng-Jun Du et al., by means of conditional random fields, realize a high-accuracy camera trajectory [76] with an average ATE of 0.030 m on the TUM dataset. The system employs graph-cut RANSAC to differentiate dynamic objects and estimate camera pose, and the static/dynamic labels are deemed the prior information of conditional random fields thereafter, so as to realize long-term observations of multiple frames. Of note, long-term observations perform strongly in low dynamic environment scenes, whereas the storage requirement increases. Recently, Zheng et al. proposed an RGB-D SLAM approach based on sub-point cloud correlations to cope with dynamic environments [77]. The map points are separated into different groups by their relative position. The system has the capacity to differentiate the dynamic object and the static portion. In addition, the real-time performance of the presented approach achieves 30.65 ms per frame, and the processing time of identifying static points only needs 2.0216 ms on average, utilizing an Intel Core i5-3470 CPU. Despite dynamic object removal resulting in a degree of increase in computational complexity, there exist benefits such as system robustness and accuracy, which is more cost-efficient. In summary, dynamic object removal based on semantics or geometry significantly improves robustness, but usually at the cost of increased computation.

#### 3.1.2. Point and Line Features

The robustness of the system in low-texture and repeat-texture scenes is an urgent need, but it is still a significant challenge for RGB-D SLAM. The repeat-texture scenes can be tackled via prior information. Kangkan Wang et al. leverage the SFM technique and a prior-based Multi-Candidates RANSAC algorithm to propose a 3D Reconstruction approach. The method is applicable to challenging outward environments with a large amount of repeated textures and significant depth missing problems [78]. The system comprises loop closure to reduce the impact of drift and is capable of inpainting the missing geometry information. The performance employs the point features approach, which is not well-suited, even though it is a mature method, and various features, such as Line Segment Detector (LSD), planar, and surfels, appear.

Unlike the conventional feature point approach, Qiang Fu et al. used a robust RGB-D SLAM method that combines point and line features to achieve high accuracy in low-texture environments with an average 29.4 fps real-time performance [79]. The LBIR algorithm has been exploited to remove outliers in the front end, and a unified model is applied to slash the reprojection error of point and line simultaneously. An RGB-D 3D reconstruction approach [80], based on the incorporation of 3D line features and dense points, is proposed. The front end of the system merges line features extracted by an efficient extractor and points; the extra constraints facilitate augmenting scan alignment, and then the performance of the entire system in a challenging environment. Concerning the back end, submap-based hierarchical optimization is leveraged to mitigate accumulated error and attain a global 3D model. The trajectory endpoint drift of the method was reduced by 22% compared with ORB-SLAM2 on the TUM dataset, but it only has around 10 fps real-time performance, leveraging an Intel Core i7-9700K CPU. Chao Sun et al. leverage hybrid point and twin line reprojection to realize an average Relative Pose Error (RPE) RMSE of 0.047 m with 32.56 ms per frame processing time on the TUM dataset [19]. In the case of some scenes, leveraging peculiar structure information is a shortcut to avoid tracking failures. Joan P. Company-Corcoles et al. presented MSC-VO, a visual odometry, extract point and line features [81]. The approach attempts to utilize structural information and Manhattan axes in the scene to improve the accuracy of pose estimation. With the aid of the regular structure peculiar to some environments, it legitimately enables the accuracy and robustness of the SLAM system to have a salient increase.

Capitalizing on distinctive features in specific scenes is also an eminent strategy to elevate the robustness of the SLAM system. Javier Gimenez et al. treat the regularity of trunks as a kind of feature, thereby presenting a SLAM that attains an appealing performance in experiments [82]. The core of the strategy sample is to perceive surroundings with the aid of structural regularities, such as the Atlanta or Manhattan world of the environment, thereby decoupling the camera rotation and alleviating the non-linearity of the system. Additionally, a tracking-by-detection scheme is reported in order to figure out the underlying structure of the environment. Nevertheless, the approach still does not have the capacity to achieve 6-DoF camera poses in that short range and viewing angles of RGB-D cameras. Eunju Jeong et al. leverage Linear Four-Point LiDAR to modify L-SLAM and expand the application range of it [83]. The usage of point and line features augmented the robustness and accuracy of the RGB-D system but increased data association challenges and computational complexity. Combining point and line features alleviates the lack of features in low-texture environments, but the extra constraints from line matching require more complex back-end optimization.

#### 3.1.3. Plane Features and Surfels

Plane features tend to persist in the scene for long durations compared to point features. They seldom disappear or undergo significant changes, making them reliable for long-term SLAM tasks and providing stable maps and pose estimates [84]. In order to resist a succession of problems that emerge from 3D plane segmentation, Zhen Dong et al. transform the 3D plane segmentation procedure into a global energy optimization problem and have an appealing performance, whether a high- or low-quality point cloud is in use [85]. The proposed method achieves high plane precision and recall in both TLS and RGB-D point clouds: 94.2% precision and 95.1% recall for TLS, and 90.4% precision and 91.4% recall for RGB-D. Wang et al. proposed a stereo visual SLAM algorithm for low-texture environments that fuses point, line, and plane features, with particular emphasis on the robust extraction and utilization of planar structures to enhance localization accuracy [86]. In case the matched plane features have no capacity to provide adequate constraints, a preferable six Degrees of Freedom (6-DoF) camera pose estimation will be realized with the STING-based scan matching method. For structured indoor environments, Yang et al. proposed PLPM-SLAM, an RGB-D SLAM framework that integrates orthogonal Manhattan plane constraints with point-line-plane joint optimization to enhance robustness and accuracy [87]. Bingjian Gong et al. introduced a plane detection and segmentation approach and utilized Planar Prior fruitfully so as to deliver high-accuracy pose estimation, thereby achieving high-quality reconstruction when operating at a frame rate of over 30 fps [88]. Moreover, a plane-based map representation has been exploited to carve out a memory footprint and reserve geometric details to the maximum extent. The map representation of plane features is relatively simple and compact, effectively reducing the storage and computational burden of the map, which balances the computational complexity introduced by the extraction of plane features. However, the representation capability of plane features is limited for non-planar terrains or objects. In environments with complex geometries or non-planar surfaces, plane features may not provide sufficiently accurate descriptions, which results in incomplete or inaccurate maps. Similarly, it should be pointed out that stable objects can be beneficial in establishing a wide range of common view relationships and effectively reducing the cumulative error of visual localization. Shiqi Lin et al. presented Contour-SLAM, an RGB-D object-level SLAM system that leverages voxels and cuboids to attain a 3D object model to substitute the general model method [89], followed by the projection errors defined by means of outer contour distance, and then utilizing projection constraints to realize state estimation. Additionally, via a covisibility graph, a local map can be reserved, and a multi-view BA can be used to optimize it. Compared to ORB-SLAM3, Contour-SLAM obtains an average ATE improvement of 72.9% in six sequences on the TUM dataset.

Surfels, which are surface elements, offer a more comprehensive representation of a scene as they incorporate not only positional information but also surface normal vectors and color information [39]. This enhanced representation enables a more precise and resilient depiction of local surface structure and appearance. This is especially beneficial when there are changes in viewpoint or variations in lighting conditions. A real-time dense visual SLAM leveraging an RGB-D camera as a sensor, dubbed SP-SLAM, was proposed by Hong et al. in 2025 [90]. By means of frame-to-model pose tracking and windowed surfel-based fusion, the approach has the capacity of capturing surfel-based maps in indoor environments without requiring post optimization. And the global consistency of surfel-based maps boils down to global loop closure. Followed by a novel light source detection method presented to improve the accuracy of pose tracking and the robustness of the entire system. The system demonstrates dense reconstruction and localization accuracy. However, it is important to note that the current implementation is limited to indoor environments. The limitation arises from the fact that the complexity of the system increases with the number of surfels in the map, even with the implementation of pose graph optimization methods. Hae Min Cho et al. presented a SLAM approach called SP-SLAM, which has an outstanding performance in low-texture environments [90]. The foremost trait of SP-SLAM treats surface elements as a kind of feature and detaches them into a hint of surfels so that the system can depict the environment and utilize a compact memory. When it comes to pose optimization, a novel BA that exploits the objective function is proposed. SP-SLAM achieves ATE RMS of 0.024 m in of/kt2n sequence on ICL-NUIM dataset. Surfels provide an approximation of the underlying 3D surface geometry using a collection of oriented discs or spheres. This approximation can result in a loss of geometric accuracy, especially for complex or highly detailed surfaces. Plane features and surfels provide more stable geometric priors than point features, but their expressiveness is limited in non-structured scenes, and they need to complement other features.

#### 3.1.4. Edge Features

Edge features provide rich geometric information, enabling the accurate representation of object boundaries and contours, which provides high-level semantic information and enhances the scene understanding capability of RGB-D SLAM systems. Moreover, the computational complexity of edge feature extraction is relatively low, ensuring real-time performance of the system. Wang et al. proposed ROEVO [91], an RGB-D visual odometry method that exclusively leverages organized edge features, enabling robust tracking and precise pose estimation through edge-level residuals and edge-level bundle adjustment. Approximate nearest neighbor fields and oriented nearest neighbors have been utilized for 3D-2D edge alignment, thereby reducing the computational requirements. Huei-Yung Lin and Jhih-Lei Hsu extract features from edge RGB-D images for pose adjustment and use a sparse visual odometry approach [92]. Followed by a posterior probability being defined for each of the edge points in order to achieve the keyframe matching procedure, thereby augmenting the accuracy of pose estimation. Experiments show the method achieving an ATE RMSE of 0.011 m in the fr2/rpy scene on the TUM dataset. In Plane-Edge-SLAM, presented by Qinxuan Sun et al., planes and edge features are fused seamlessly by means of an adaptive weighting algorithm [93]. A probabilistic plane fitting algorithm has been explored to fit plane models to noisy 3D points so as to augment the accuracy and robustness of the entire system remarkably. However, it is significant to consider and address the disadvantages of edge features in practical applications, such as sensitivity to occlusion, high susceptibility to noise, and the requirement for sufficient texture. Edge features are computationally efficient and geometrically rich, yet sensitive to occlusion and noise—suitable for fast motion in texture-repetitive scenes.

#### 3.1.5. Semantic Information and Deep Learning

Even though great accomplishments have been made by the usage of feature extraction in the front end, it still has various flaws, such as noticeable performance degradation in low-texture environments and sensitivity to ambient light and texture. One option is to use neural networks to attain semantic information, thereby estimating the camera pose independently or with geometric information [64]. By leveraging large-scale training data and powerful feature learning capabilities, these methods exhibit strong robustness [28] to factors such as different lighting conditions, background interference, and pose variations. This allows RGB-D SLAM systems to recognize and track moving objects in complex environments at a human level, thereby improving system stability and robustness. Mask-RCNN is a deep learning-based method for object detection and instance segmentation, which combines object detection with pixel-level segmentation tasks. It emphasizes accuracy over running speed. In the context of RGB-D SLAM, Mask-RCNN can be applied for dynamic object detection and tracking, enhancing the perception and mapping capabilities of the system. Ao Li et al. proposed DP-SLAM based on a moving probability propagation model and by means of the combination of geometry constraints and semantic segmentation to track dynamic keypoints [94]. The whole method can be utilized in the front end of ORB-SLAM2 to enable the system to get a better handle on the dynamic environment, and the average RMSE improvement values for ATE, translational drift, and rotational drift of RPE are 95.75%, 63.11%, and 46.29%, respectively. A motion detection and segmentation technique, introduced by Wanfang Xie et al., has been explored to improve ORB-SLAM2 so as to achieve high-accuracy localization in a dynamic environment [95]. The foremost trait of the approach is to detect and segment active dynamic objects by means of a mask inpainting method, whereas detecting passive dynamic objects via LK optical flow. However, the processing time per frame when using this method increases to 1.1 s, which is a formidable problem. On the foundation of ORB-SLAM2 [95], a tightly-coupled hybrid detection algorithm that combines semantic and geometric information has been explored for detecting dynamic objects. Subsequently, feature point weights are defined and utilized on dynamic information so as to transform pose optimization into weight-based joint optimization. Similarly, the method needs an extra average of 256.87 ms per frame of processing time compared with ORB-SLAM. Berta Bescos et al., by means of multi-view geometry and Mask R-CNN, realize high accuracy dynamic object detection in the RGB-D camera case [96]. With the aid of the position of the camera, the system is capable of inpainting the background that is occluded by dynamic obstacles. On the foundation of DynaSLAM, Berta Bescos et al. proposed an object-level SLAM dubbed DynaSLAM II [30]. Similar to DynaSLAM, DynaSLAM II leverages semantic segmentation and ORB features to detect and track dynamic objects in scenarios. A distinctive feature it has is optimized scene structure, and the trajectories, both camera and dynamic objects, capitalize on a novel BA approach. It is worth noting that camera tracking and dynamic tracking are mutually beneficial. DynaSLAM II furnishes about 10 fps real-time performance when facing 20 objects at a time. Based on DynaSLAM, Zihao Pan et al. proposed a 3D reconstruction algorithm that has the capacity of mapping in a dynamic environment [97]. The algorithm re-extracts feature points in order to solve the problem of scarce original feature points, and the image is segmented by Mask R-CNN. Additionally, PCA is utilized in the post-processing of point clouds and aims at lessening noise substantially, while a filtering approach capitalizes on slashing dynamic outliers.

A robust semantic RGB-D SLAM dubbed RS-SLAM is presented by Teng Ran et al., which leverages a semantic segmentation model to detect not only dynamic objects but also potential moving objects simultaneously [75]. Moreover, an updating technique enables the system to generate a static semantic OctoMap in a dynamic environment. RS-SLAM obtains an average RMSE improvement for ATE of 91.49% and a Standard Deviation improvement of 88.03% compared with ORB-SLAM2 on the TUM RGB-D dataset. An approach that can run robustly in a dynamic environment is called Blitz SLAM [75]. The geometric and semantic information is applied to eliminate the dynamic region in the local point cloud map, which can undermine the process of mapping. Furthermore, a series of local maps combine to form the global map. The average RMSE improvements on the TUM RGB-D dataset for ATE, RPE, and RPE in dynamic sequences are 95.64%, 92.29%, and 89.62% compared with ORB-SLAM2, respectively. Through dual decoupling of semantic and geometric priors, Chen et al. achieve accurate 3D scene completion, the foremost achievement of this research, and attain state-of-the-art performance in both semantic and geometric metrics on autonomous driving benchmarks [98].

Bearing a resemblance to Mask-RCNN, PSPNet, ResNet-50, and 2D-CNN, which also focus on accuracy, require high computational resources from the system. On the other hand, the YOLACT and NCNN networks emphasize real-time performance. Jianfang Chang et al. leverage the real-time instance segmentation network dubbed YOLACT so as to result in a prescription of detecting dynamic objects and elevate the robustness of the SLAM system [99]. Moreover, geometric constraints have been exploited to filter the feature points on dynamic objects beyond the mask and compensate for YOLACT to capitalize on dense optical flow in the RGB-D case. The segmentation speed of YOLACT is 32 ms per frame with NVIDIA RTX2080. Xiqi Wang et al. developed an improvement method of ORB-SLAM2 front-end rendering to augment the accuracy, and the entire system is devised in two key steps [100]. A missed detection compensation algorithm, utilizing the constant velocity model and region growing algorithm, has been explored. Nevertheless, there are dynamic objects that cannot be recognized yet. Accordingly, a geometric constraint model is applied to further differentiate and extract the dynamic feature points. Shuhong Cheng et al. modified the ORB-SLAM2 framework with an NCNN-based fast dynamic feature rejection algorithm and two novel parallel threads, which are an object detecting thread and a semantic object map mapping thread, respectively [101].

Additionally, Gaurav Singh et al. presented a motion state detection method, via the usage of depth and feature flow information, to signify the region with moving probability, thereby fused with semantic information to achieve high-quality dynamic object detection [102]. For the sake of computational complexity, the approach extracts semantics on keyframes only with principal changes. Experiments signify that the method applied to ORBSLAM2 has a lower per-frame processing time of 0.215 s compared to using it in ORBSLAM3 with 0.235 s on Jetson TX1. Shenghao Li et al. leverage a joint feature detection and description network, trained by a self-supervised learning method iteratively, to extract quantized local features [103]. The approach endows the entire system with the capacity of transforming environmental fluctuation into a trivial impact and reap conspicuous improvement in experiments on accuracy and robustness. RGB-D SLAM is amenable to practical applications with the aid of deep learning. Hengli Wang et al. presented a self-supervised learning method for robotic wheelchairs, which is capable of detecting drivable areas and road anomalies [104]. With the aid of an RGB-D camera and a SLAM-based coordinate system [63], Yao Guo et al. proposed a system capable of tracking and analyzing the gait of humans. When referring to active RGB-D SLAM, Xu-Yang Dai et al. modify ORB-SLAM2 to reconstruct a three-channel navigation map for robots, thereby presenting an active RGB-D SLAM framework [105]. The entire system is composed of two core components in this valuable work. The former is an Active-Neural-SLAM (ANS) model that treats human searching behaviors as directions, enabling robots to explore indoor environments at a human level. The latter is a kind of RGB-D camera view planning policy trained by generative adversarial imitation learning (GAIL), capable of actively adjusting the camera view. Under various experiments, the proposed active RGB-D SLAM brings significant improvements in exploration coverage ratio for unknown environments. On average, there is a 53.08% increase in coverage compared to traditional methods, and the number of effective exploration steps before tracking failure occurs has a remarkable increase of 405%. Deep learning injects high-level understanding into the front end, showing advantages in dynamic environments and active exploration, but the trade-off between real-time performance and computational resources remains.

### 3.2. Neural Implicit/Dense RGB-D SLAM

Direct methods exploit every pixel in the image instead of a sparse set of feature points, yielding denser and more robust visual information that is especially advantageous in low-texture, repetitive, or feature-poor environments [44]. Because they bypass explicit feature extraction and matching, direct approaches often achieve higher computational efficiency and strong real-time performance [106]. However, their performance can degrade noticeably under dynamic occlusions or when the limited field of view of an RGB-D camera restricts scene coverage.

To address these issues, Zikang Yuan et al. proposed RGB-D DSO, a direct sparse visual odometry that combines sliding-window optimization with a dedicated depth refinement module; experiments showed it runs 1.4× faster than ORB-SLAM2 on an Intel i7 CPU [107]. Deok-Hwa Kim and Jong-Hwan Kim introduced BaMVO, an energy-based dense visual odometry that models the background non-parametrically, achieving an average processing time of 24.86 ms per frame on an Intel i7 in dynamic scenes [108]. To handle illumination changes, Lee Clement and Jonathan Kelly designed a deep convolutional encoder-decoder that transforms incoming RGB-D images to match the lighting conditions of previous frames, thereby strengthening the reliability of direct VO systems [109].

Building on the philosophy of pixel-level information usage, a new family of dense RGB-D SLAM systems has emerged that leverages neural implicit representations to simultaneously reconstruct geometry and appearance with high fidelity. Table 2 summarizes the performance of six recent neural implicit/dense SLAM methods on the Replica dataset, all evaluated on a platform equipped with an Intel Core i9-12900HX CPU and an NVIDIA RTX 3080ti 16 GB Laptop GPU. Photo-SLAM [110] takes a hybrid geometric-photometric approach without any learned neural network, achieving an average RMSE of 0.590 cm and a per-frame processing time of 48.55 ms. Orbeez-SLAM [111] couples ORB geometric features with a NeRF-based mapping module built on instant-ngp, yielding an RMSE of 0.888 cm at 24.19 ms. ESLAM [112] employs multi-scale tri-plane neural features decoded by separate MLPs for geometry and appearance, attaining a leading RMSE of 0.568 cm but requiring a longer 149.54 ms per frame. Co-SLAM [113] relies on hash-grid and coordinate encodings with a geometry decoder and a color MLP, resulting in 1.158 cm RMSE and 68.61 ms per frame. Go-SLAM [114] extracts learned geometric features from optical flow and uses a hash-encoded SDF MLP alongside a color MLP, with an RMSE of 0.571 cm and a processing time of 51.48 ms. Point-SLAM [115] adopts a point-based neural scene representation, delivering 0.596 cm RMSE but at a substantially higher cost of 2898.55 ms per frame, clearly illustrating the trade-off between reconstruction accuracy and computational efficiency. Collectively, these works demonstrate that neural implicit representations can push dense RGB-D SLAM toward highly accurate, dense mapping, while ongoing research continues to close the gap between precision and real-time performance. Neural implicit representations push the accuracy boundary of dense reconstruction; however, their computational cost is still much higher than that of traditional feature-based methods—a classic accuracy-speed trade-off.

In comparison, ESLAM achieves the highest accuracy (ATE RMSE 0.568 cm) among all frameworks, while Orbeez-SLAM offers the fastest processing time (24.19 ms) with moderate precision.

Point-SLAM delivers competitive accuracy (0.596 cm) but suffers from high processing time (2898.55 ms), making it impractical for real-time applications.

### 3.3. Hybrid Method

Besides feature-based and direct approaches, a hybrid strategy combines several methods aforementioned, termed the semi-direct approach [45]. SID-SLAM is the first tightly-coupled semi-direct RGB-D SLAM that utilizes an RGB-D camera as a sensor independently [116], which makes use of covariance models and information-based processes for the selection of informative points and keyframes. Information metrics have been exploited to lessen the state size without sacrificing accuracy, and the experiment shows that the system provides cutting-edge accuracy and robustness. An RGB-D Visual Odometry presented by Hang Luo et al. conducts joint tracking and windowed BA, capitalizing on the combination of feature-based and direct approaches [117]. Followed by the mapping accuracy being improved via the usage of a slanted plane model. The proposed method demonstrates average absolute trajectory root-mean-square errors of 1.50 cm, 1.44 cm, and 1.74 cm on ICL-NUIM, TUM RGB-D, and ETH SLAM datasets, respectively.

In order to prohibit noticeable performance degradation in low-texture and light-persistent fluctuation environments, Jiyuan Cai et al. presented E-DVO, a Direct RGB-D VO, aiming at achieving outstanding high accuracy and robustness in the above situations [92]. The prescription of E-DVO is to utilize a bi-directional framework based on edge features. In addition, the drift arising from long-term operation is alleviated by means of a switching strategy. E-DVO shows the average RMSE of translational RPE of 0.0294 m on the TUM RGB-D dataset. Cong et al. proposed a human–robot collaborative mapping framework named HRCM [63], which tightly couples a lightweight LiDAR-inertial system on a manned helmet with an unmanned ground vehicle platform, enabling collaborative SLAM across heterogeneous agents for intelligent joint exploration. The front end uses a semi-direct approach and a deep learning method for object detection in RGB-D input images, ensuring that the selected feature points are defensible. The performance comparison of representative RGB-D SLAM leveraging multifarious methods is shown in Table 2. Semi-direct methods combine the advantages of feature-based and direct approaches, achieving more stable performance in low-texture and varying-lighting scenes, albeit with higher implementation complexity.

### 3.4. Multi-Sensor Fusion

RGB-D cameras have inherent limitations and drawbacks. They generally have a narrow field of view (FOV) compared to regular RGB cameras, which can be a challenge when sampling to capture a wide or panoramic scene [118]. In addition, the decrease in depth accuracy at the edges and the limited range for depth sensing also cloud the usage of RGB-D SLAM [119]. Multi-sensor fusion is termed as a significant means to overcome the inherent limitations of diverse sensors so as to achieve robustness and accuracy performance in challenging environments. However, the excess data provided by several sensors invite computational burden, and the cost of the system has increased evidently. The performance comparison of various multi-sensor fusion RGB-D SLAM is demonstrated in Table 3. When it comes to the FOV, Kolla and Vundavilli fuse wheel odometry, an RGB-D camera, and an IMU within the RTAB-Map framework to estimate the information of localization [120]. The proposed dual RGB-D cameras strategy explores a 31.0 m^2^ area in 196 s, whereas the method leveraging only one camera explores 27.6 m^2^ using 297 s. In relation to the issue of large-scale indoor scenarios reconstruction, Wang et al. proposed PLGSLAM [121], a neural visual SLAM system that utilizes a progressive scene representation to dynamically allocate local neural fields within a sliding window, enabling high-fidelity surface reconstruction and robust camera tracking across expansive indoor environments. Especially in terms of sensor acquisition, the approach leverages multiple unsynchronized RGB-D cameras as sensors, and a tracking algorithm is presented in order to realize camera tracking simultaneously. In the process of integrating original frames into a panorama, uncertainty estimates are obtained so as to preferably create a model of sensor noise and augment geometric quality in the panorama. Experiments support the approach, achieving reconstruction quality with an ATE RMSE of 5.16 mm in the first scan.

A combination of RGB-D cameras and an Inertial Measurement Unit (IMU), the RGB-D system offers several advantages. IMU aids in compensating for camera pose, improving localization and mapping accuracy. It also provides motion estimation, enhancing the ability of the system to estimate camera movement. This integration reduces reliance on depth information, increasing system robustness. Moreover, the high update rate of IMU improves real-time performance, enabling faster localization and mapping. Overall, this sensor fusion approach enhances the accuracy and robustness capabilities of SLAM systems. Freißmuth et al. presented a real-time mapping and analysis system that harnesses a pose graph LiDAR SLAM framework to conduct forest inventory tasks, and the results demonstrate that it is capable of providing accurate and robust prescriptions for forest measurements, achieving a DBH estimation bias of only 1.93 cm across conifer, broad-leaf, and mixed forests [122]. Chengbing Chu and Shidong Yang et al. utilize an Extended Kalman Filter (EKF) to fuse information from two types of sensors and demonstrate the capability to achieve the calibration of gravity and camera extrinsic, and the error ratios of the method are only 0.57% in an office scene [123,124].

On the foundation of ORB-SLAM and ORB-SLAM2, Bui et al. proposed SuperPoint-SLAM3 [125], a drop-in upgrade that replaces the ORB front end with a self-supervised deep feature detector and introduces NetVLAD-based place recognition for learning-based loop closure. Wang et al. proposed Stereo-NEC [126], an enhanced stereo visual-inertial SLAM initialization approach that first independently estimates the gyroscope bias and formulates a maximum a posteriori (MAP) problem for further refinement to estimate scale, gravity direction, and the remaining IMU biases, outperforming ORB-SLAM3 in accuracy and robustness on the EuRoC benchmark. Of special note is that an improved place recognition algorithm with high recall is introduced to detect loop closures. Experiments show that ORB-SLAM3 achieves high accuracy, especially being 2 to 10 times more accurate in visual-inertial mode, which exerts enormous influence in the realm of VSLAM. It obtains an accuracy of 3.5 cm on the EuRoC drone and 9 mm on the TUM-VI facing swift camera motion. Zhanghao Zhu et al. proposed a visual-inertial mode tightly coupled with encoders, leveraging a generative measurement model to resist the temporary failure of pure VSLAM [127]. Additionally, the article derives an expression of maximum a posteriori estimation and Jacobians for optimization. For resource-restricted robots, a real-time RGB-D SLAM system named SDT-SLAM [128] was proposed, which integrates object detection and feature tracking simultaneously as its core components. The depth information from the RGB-D camera has been utilized for dynamic feature recognition, and the IMU is used in feature tracking and consistency checking.

Line features, plane features, specific structure, and semantic information have also been utilized in VIO. E. Jared Shamwell et al. originally proposed VIOLearner (2018), which learns to estimate absolute trajectories and generate hypothesis trajectories from inertial and RGB-D imagery without sensor calibration. More recently, Rajvanshi et al. presented ZeroVO [129], a visual odometry algorithm that achieves zero-shot generalization and robustness to uncalibrated setups, extending this line of research with semi-supervised learning. The principal component of it is online error correction (OEC) modules, which correct hypothesis trajectories and achieve high-accuracy trajectory estimates. Jie Xu et al. presented R^2^DIO, which utilizes agglomerative hierarchical clustering (AHC) to extract 3D line and plane features efficiently and integrate multimodal constraints [130]. Directional consistency has been used to filter mismatches in the process of feature alignment. Furthermore, multimodal constraints, including line and plane matching constraints, inertial measurement unit pre-integration constraints, and historical odometry constraints, are applied to generate a dense map. Applying You Only Look Once (YOLO) in SLAM offers the advantage of accurate object detection and tracking. It enables simultaneous perception and mapping, enhancing the ability to handle dynamic environments efficiently. Zhanyuan Chang et al. modified ORG-SLAM3 by leveraging the YOLOv4 tiny network to resist the decline of accuracy influenced by dynamic objects and fuse point and surface features to back camera pose tracking [131]. The entire system circumvents the limitation of the conventional feature-based method, thereby having a conspicuous average accuracy lift of 94.08% compared with ORB-SLAM3. Taking advantage of multi-sensor measurements, heterogeneous landmarks, and the regularity of the environment detected by an Atlanta world inference approach, a tightly-coupled RGB-D inertial odometry called S-VIO has an appealing performance in terms of accuracy and robustness [132]. Followed by a real-time RGB-D SLAM dubbed OVD-SLAM, presented by Jiaming He et al., which utilizes multiple information comprising semantic information, depth information, and optical flow to differentiate foreground, background, and recognize dynamic regions [133]. As a last resort, an optimization weight is applied to lessen the impact of dynamic objects at each point in pose optimization. OVD-SLAM achieves impressive results on the TUM RGB-D dataset, with an ATE RMSE of less than 0.035 m. Additionally, it demonstrates excellent real-time performance, with an average per-frame tracking time of less than 30 ms using an i7 CPU and RTX 2060 GPU.

The multi-sensor fusion SLAM methods are exhibiting a trend toward diversification driven by the increasing availability and advancements in various sensor technologies [134]. This diversification trend allows for tailored sensor combinations based on specific application requirements, leading to versatile and effective SLAM solutions. Mathieu Labbé and François Michaud extend RTAB-MAP, an open-source library that has comprehensive applications in the realm of SLAM, on the previous foundation, enabling the SLAM system to support both VSLAM and LiDAR-based SLAM [126]. As a consequence, the system has preferable flexibility and fulfills the requirement of comparing and selecting appropriate prescriptions for distinct scenarios and applications. A tightly coupled visual-inertial odometry dubbed VDIWO was presented by Xinyang Zhao et al. [135], which has been explored for depth information appropriately. VDIWO fused RGB-D camera, IMU, and wheel odometry into a tightly coupled optimization framework and achieved accurate localization both in indoor and outdoor environments. Visible light positioning (VLP) has the capacity to support high-accuracy localization in indoor environments, making it a future direction to be explored in SLAM. VWR-SLAM, proposed by Junlin Huang et al., is a multi-sensor fusion SLAM framework that tightly couples several sensors in terms of visible light positioning, wheel odometer, and RGB-D camera [136]. The system generates a 3D sparse map and VLP-landmark map simultaneously and then realizes high accuracy and robust SLAM despite the environment only having a hint of light. It achieves an average robot positioning accuracy of 1.81 cm and an LED mapping accuracy of 3.01 cm.

**Table 3:** Performance comparison of multi-sensor fusion RGB-D SLAM.

Framework	Sensors	Dataset/Sequence	ATE RMSE (m)	Processor	Average Process Time (ms)
ORB-SLAM3 [137]	IMU+ RGB-D Camera	AR/VR scenarios/room6	0.006	Intel Core i7-7700 CPU	30–40
VIEOS2 [138]	IMU+ Encoder + RGB-D Camera	Indoor Corridor (15 Hz frame) (200 Hz Encoder, IMU Data)	0.029	-	25
Dynamic-VINS [139]	IMU+ RGB-D Camera	OpenLORIS-Scene/market	0.012	NVIDIA Jetson AGX Xavier	43.04
VIOLearner [139]	IMU+ RGB-D Camera	KITII/09	0.042	Intel Core i7-6950X CPU NVIDIA Titan X GPU	27
R^2^DIO [130]	IMU+ RGB-D Camera	Exhibition hall I	0.011	Intel i5-1137G7 CPU	<40
S-VIO [132]	IMU+ RGB-D Camera	OpenLORIS-Scene/corridor	0.024	Intel Core i9 CPU	<30
VDIWO [135]	IMU+ Wheel Odometry RGB-D Camera	OpenLORIS-Scene/Home1-5	0.152	Intel NUC i7-1165 CPU	-

To systematically examine the landscape of modern visual SLAM, we contrast five prominent paradigm categories: feature-based methods, direct/dense methods, neural implicit SLAM, semi-direct/hybrid approaches, and multi-sensor fusion SLAM. Each category exhibits distinct trade-offs in accuracy, real-time capability, robustness, and computational demand. Table 4 summarizes their core advantages, limitations, typical application scenarios, and quantitative performance profiles. The evaluation data underpinning this comparison are drawn from typical experimental setups widely adopted in the SLAM literature: standardized benchmarking datasets such as EuRoC MAV, TUM RGB-D, and KITTI are used for pose accuracy (measured by absolute trajectory error ATE or relative pose error RPE). Real-time performance and computational cost are assessed on representative hardware, ranging from embedded platforms (e.g., ARM Cortex-A series, Intel Core i7-9700K CPU) for lightweight algorithms, to workstation-class configurations with NVIDIA RTX 3090 GPUs for neural and dense methods. Robustness is evaluated under challenging real-world conditions, including rapid motion, illumination variation, and sensor degradation.

Multi-sensor fusion effectively overcomes the limited field of view, range, and dynamic challenges of a single RGB-D camera, but system cost and calibration complexity also increase, a trade-off to be made according to the application scenario.

## 4. Discussion

From the comparative analysis of the reviewed RGB-D SLAM approaches, three key findings emerge: feature-based methods are mature and efficient but fail in low-texture or dynamic scenes; direct and semi-direct methods improve robustness under challenging conditions at the cost of sensitivity to illumination changes; learning-based and neural implicit approaches achieve state-of-the-art accuracy and dense reconstruction but demand high computational resources. A deeper cross-method comparison reveals that no single technique dominates all scenarios. Feature-based methods excel in structured, texture-rich environments; direct methods are preferable when feature extraction fails; semantic and multi-sensor fusion methods are necessary for dynamic or large-scale scenes; neural implicit SLAM remains impractical for real-time mobile robotics. The central performance trade-offs are between accuracy and real-time performance, and between robustness and computational cost. High accuracy often requires dense processing or deep networks, which increase latency and memory, while lightweight methods sacrifice accuracy under difficult conditions. For practitioners, application-oriented guidelines can be summarized: use ORB-SLAM2/3 for indoor static environments; adopt point-line or direct methods for low-texture scenes; employ semantic SLAM (e.g., Dyna SLAM II) for dynamic environments; integrate IMU or LiDAR for large-scale or outdoor scenarios; recent advances along this line can be found in [140,141,142,143]. And avoid neural implicit methods on resource-constrained platforms. Finally, current challenges in real-world deployment include handling arbitrary dynamic objects without prior knowledge, maintaining robustness under lighting and weather changes, ensuring long-term operation with reliable loop closure and map maintenance, balancing computational efficiency on embedded devices, and overcoming sensor limitations such as restricted depth range, narrow field of view, and sensitivity to reflective surfaces. Addressing these challenges will guide future RGB-D SLAM research.

## 5. Conclusions

RGB-D SLAM has emerged as a significant research direction because it can jointly utilize appearance and depth information to improve localization and mapping performance. In this review, we systematically summarized the RGB-D SLAM pipeline, including the sensor layer, front-end visual odometry, back-end optimization, loop closure detection, and mapping. Representative methods under the feature-based, direct, and learning-based paradigms were also reviewed, together with their respective strengths and limitations in different application scenarios.

Overall, RGB-D SLAM offers distinct advantages in dense reconstruction, scale consistency, and scene interpretation. However, several critical challenges remain unresolved in practical deployment, including sensitivity to dynamic objects, illumination variation, low-texture environments, motion blur, limited depth range, and computational burden. On the basis of our findings, it can be concluded that RGB-D SLAM tends to prioritize robustness and accuracy, even at the expense of real-time performance or increased cost, when applied in challenging environments. Failures caused by ambient influences and inaccuracies are more severe than mere time slowdowns or high costs. Consequently, the current research focus has shifted from pursuing isolated metric improvements to achieving a better trade-off among multiple requirements in specific application contexts. Thus, performance trade-offs oriented to real-world requirements have become the central theme of RGB-D SLAM research, replacing the pursuit of a single metric.

## 6. Outlook

Future RGB-D SLAM research is expected to move toward deeper integration with artificial intelligence, particularly in semantic perception, object-level understanding, and intelligent map construction. Compared with purely geometric approaches, AI-enabled methods can provide richer scene representation, support object recognition, and improve discrimination between dynamic and static regions, thereby enhancing robustness in complex environments. In this regard, semantic SLAM and object-aware mapping are likely to become increasingly important research directions.

Meanwhile, several unresolved scientific issues still warrant systematic investigation. First, RGB-D SLAM must become more robust in dynamic, unstructured, and low-texture environments, where feature extraction and geometric consistency remain unstable. Second, the inherent trade-off among accuracy, real-time performance, memory consumption, and energy efficiency continues to be a major bottleneck, especially for embedded and mobile platforms. Third, long-term autonomy still depends on more reliable loop closure, relocalization, and map maintenance mechanisms to suppress drift and preserve global consistency over extended operation. These problems are not merely implementation details; they are central to the transition of RGB-D SLAM from controlled laboratory settings to dependable real-world applications.

In addition, future systems may benefit from more effective multimodal fusion and more lightweight network architectures. Integrating RGB-D cameras with inertial sensors or other complementary modalities can improve robustness under rapid motion, illumination changes, and partial occlusions. At the same time, efficient AI models should be designed to support real-time deployment without excessive computational overhead. More broadly, the next stage of RGB-D SLAM should aim not only at more accurate pose estimation and denser mapping, but also at more intelligent scene representation, stronger adaptability, and a deeper understanding of the surrounding environment.

## Figures and Tables

**Table 1:** Cameras applied to RGB-D SLAM.

Sensor	Frame Rate (Hz)	Measurement Range (m)	Price (€)	Power Consumption (W)
TOF RGB-D Camera	10–400	0.1–40	>38	1–5
Structured Light RGB-D Camera	10–120	0.01–10	>150	<5
Stereo Camera	1–400	0.1–200	400–4K	2–15

**Table 2:** Performance comparison of neural implicit and dense RGB-D SLAM methods.

Framework	Features	Neural Network	Dataset/Sequence	ATE RMSE (cm)	Processor	Average Process Time (ms)
Photo-SLAM [110]	Hybrid geometric + photometric	No Have	Replica/Avg.	0.590	Intel Core i9-12900HX CPU NVIDIA RTX 3080ti 16 GB Laptop GPU	48.55
Orbeez-SLAM [111]	ORB geometric features + NeRF mapping	NeRF (instant-ngp)	Replica/Avg.	0.888	Intel Core i9-12900HX CPU NVIDIA RTX 3080ti 16 GB Laptop GPU	24.19
ESLAM [112]	Multi-scale tri-plane neural features	MLP decoders (geometry + appearance)	Replica/Avg.	0.568	Intel Core i9-12900HX CPU NVIDIA RTX 3080ti 16 GB Laptop GPU	149.54
Co-SLAM [113]	Hash-grid + coordinate encoding features	Geometry decoder + color MLP	Replica/Avg.	1.158	Intel Core i9-12900HX CPU NVIDIA RTX 3080ti 16 GB Laptop GPU	68.61
Go-SLAM [114]	Learned geometric features (flow-based)	Hash-encoded SDF MLP + color MLP	Replica/Avg.	0.571	Intel Core i9-12900HX CPU NVIDIA RTX 3080ti 16 GB Laptop GPU	51.48
Point-SLAM [115]	Point-based geometric + neural features	Point-based neural scene representation	Replica/Avg.	0.596	Intel Core i9-12900HX CPU NVIDIA RTX 3080ti 16 GB Laptop GPU	2898.55

**Table 4:** Comparison of five mainstream SLAM paradigms.

Method Category	Advantages	Limitations	Application Scenarios	Accuracy	Real-Time Performance
Feature-Based Methods	1. Invariant to illumination and viewpoint changes 2. Mature and stable, backed by long-term research 3. Supports loop closure detection and relocalization 4. Runs efficiently on CPU	1. Heavily reliant on texture/corners; fails in texture-less or repetitive scenes 2. Feature extraction/matching can be a bottleneck 3. Only builds sparse maps, unsuitable for dense obstacle avoidance or interaction	Medium-to-large scale indoor/outdoor localization AR navigation, mobile robots Autonomous driving VO (e.g., ORB-SLAM3)	Pose: medium–high (cm-level) Map: sparse	High (>30 fps common on CPU)
Direct/Dense Methods	1. Directly uses pixel intensity; works in texture-poor regions without feature extraction 2. Can produce dense or semi-dense maps for reconstruction and obstacle avoidance 3. Achieves high pose estimation accuracy through fine photometric alignment	1. Sensitive to illumination changes, exposure, and photometric calibration 2. Loop closure detection is difficult; prone to accumulated drift 3. Dense reconstruction is extremely compute-intensive; hard to run in real time on common hardware 4. Requires good initialization and small-motion assumption	Handheld dense reconstruction (DTAM) Small object/scene scanning UAV small-range obstacle avoidance Sparse direct (DSO) for fast odometry	Pose: medium–high Dense geometry: high (but not metric scale)	Medium–High: sparse direct (DSO) can reach 100+ fps; dense requires GPU for barely real time
Neural Implicit SLAM	1. Creates continuous, high-fidelity scene representations (radiance fields, SDF) 2. Compact maps; supports novel view synthesis and high-quality mesh reconstruction 3. Can learn complex optical properties, capturing fine details	1. Training/optimization is slow; most methods are not yet real-time 2. Extremely high compute and memory requirements (high-end GPU) 3. Poor cross-scene generalization; performance depends on thorough scene coverage 4. Loop closure and global correction immature; sensitive to dynamic elements	Small-scale, high-fidelity reconstruction VR/AR content creation, inverse rendering Surgical robots, endoscopic 3D imaging (research stage)	Reconstruction geometry: extremely high (sub-mm) Pose: medium–high (limited by network capacity)	Low–Medium: mainstream methods run 1–10 Hz or offline; recent work reaches near real time (>10 fps) with powerful GPU
Semi-direct/Hybrid Methods	1. Combines the strengths of feature-based and direct approaches; computationally efficient 2. Uses sparse direct alignment for fast pose estimation, then feature-based mapping 3. High framerate, low resource consumption	1. Can still fail during pure rotation or in texture-scarce scenes 2. Loop closure usually requires an additional module 3. Accuracy and robustness typically sit between pure feature-based and direct methods	Fast UAV navigation (e.g., SVO) Lightweight SLAM on resource-constrained systems Low-latency AR applications	Pose: medium–high (slightly below finely-tuned feature methods, but very efficient)	Extremely High (up to 100–400 Hz, e.g., SVO 2.0)
Multi-Sensor Fusion SLAM	1. Fuses IMU, LiDAR, GPS, wheel odometry, etc., compensating for single-sensor weaknesses 2. High robustness: handles darkness, texture-less scenes, high-speed motion 3. Provides absolute metric scale and high-frequency pose output 4. High accuracy and strong resistance to long-term drift	1. Complex multi-sensor calibration; strict time synchronization is mandatory 2. Increased hardware cost and system complexity 3. Needs to process heterogeneous data streams; diverse failure modes 4. Some sensors (e.g., GPS) fail indoors or under occlusion	Autonomous driving (camera-LiDAR-IMU) UAV all-source navigation Long-term autonomous mobile robot operation Smartphone AR (VIO, e.g., ARKit/ARCore)	High (globally/locally consistent cm-level, excellent short-term accuracy with IMU)	High (IMU can predict at 100–1000 Hz; visual output at 30+ fps)

## Data Availability

No new data were created or analyzed in this study.
